# Mechanisms of Cd-Induced Cytotoxicity in Normal Human Skin Keratinocytes: Implication for Human Health

**DOI:** 10.3390/ijms231911767

**Published:** 2022-10-04

**Authors:** Jing-Ya Li, Dao-Lei Cui, Yu-Mei Xie, Jin-Zhou Su, Meng-Yan Zhang, You-Ya Niu, Ping Xiang

**Affiliations:** 1 Yunnan Province Innovative Research Team of Environmental Pollution, Food Safety, and Human Health, Institute of Environmental Remediation and Human Health, School of Ecology and Environment, Southwest Forestry University, Kunming 650224, China; 2 School of Basic Medical Sciences, Hunan University of Medicine, Huaihua 418000, China

**Keywords:** cadmium (Cd), human skin keratinocytes, DNA damage, cell cycle arrest, cell apoptosis, gene expression

## Abstract

Cadmium (Cd) is one of the toxic heavy metals found widely in the environment. Skin is an important target organ of Cd exposure. However, the adverse effects of Cd on human skin are still not well known. In this study, normal human skin keratinocytes (HaCaT cells) were studied for changes in cell viability, morphology, DNA damage, cycle, apoptosis, and the expression of endoplasmic reticulum (ER) stress-related genes (*XBP-1*, *BiP*, *ATF-4*, and *CHOP*) after exposure to Cd for 24 h. We found that Cd decreased cell viability in a concentration-dependent manner, with a median lethal concentration (LC_50_) of 11 µM. DNA damage induction was evidenced by upregulation of the level of γ-H2AX. Furthermore, Cd induced G0/G1 phase cell cycle arrest and apoptosis in a dose-dependent manner and upregulated the mRNA levels of ER stress biomarker genes (*XBP-1*, *BiP*, *ATF4*, and *CHOP*). Taken together, our results showed that Cd induced cytotoxicity and DNA damage in HaCaT cells, eventually resulting in cell cycle arrest in the G0/G1 phase and apoptosis. In addition, ER stress may be involved in Cd-induced HaCaT apoptosis. Our data imply the importance of reducing Cd pollution in the environment to reduce its adverse impacts on human skin.

## 1. Introduction

With the accelerated development of industrialization and urbanization processes, the heavy metal burden in the environment is gradually increasing [1]. Among heavy metals, cadmium (Cd) is widely distributed in the environment [2,3,4]. Cd is a persistent environmental toxicant with a long half-life: it cannot be degraded by microorganisms; has a certain degree of bioaccumulation in soil, animals, and humans; and, through the entry of Cd-contaminated soil, food, and dust into the body, harms human health [5]. It has been reported that the Cd content in dust was 0.25–14.5 mg/kg [6,7]. Moreover, it was also detected in the liver (60–62 μg/g), pancreas (40–50 μg/g), and thyroid (35–45μg/g) of humans who live in contaminated areas [8]. Cd has been classified as a Group I carcinogen by the IARC (International Agency for Research on Cancer), indicating that it poses a great threat to the health of humans [9]. Uptake by humans occurs mainly through exposure to Cd-contaminated soil, dust, and cigarette smoke, from which multiple organ dysfunction can be observed in the organisms [10,11]. For example, chronic exposure to large amounts of Cd can trigger clinical renal Fanconi syndrome, which can eventually lead to kidney failure [12]. Exposure to environmental Cd is associated with necrotizing inflammation of the liver [13]. In addition to dietary intake and inhalation, skin absorption is also an important route of Cd exposure. Thus, it is a known fact that Cd absorption can cause significant damage to the human body. Wang et al. [14] reported that skin absorption may be the main route for human exposure to heavy metals, including Cd. This is because the skin is the body’s first line of defense and the largest organ of the human body. However, little is known about the Cd-induced adverse effects on human skin.

Numerous studies have shown that Cd induced various adverse effects on biological systems [15,16]. Studies have proved that Cd causes prostate cancer in rodents, triggers reproductive disorders in males and females, and decreases fertility [17,18,19,20]. Huang et al. (2021) suggested that Cd exposure regulates nerve cell proliferation and death, and induces neuroinflammation, which affects the normal development of the fetal brain [21]. In addition, the bactericidal potential of microbially synthesized Cd oxide nanoparticles causes oxidative stress and protein leakage in bacterial cells [22,23,24]. Wang et al. (2021) reported that Cd induced oxidative damage and cell apoptosis, and triggered cell cycle arrest in human gastric epithelial cells [25]. The cell cycle is a rigorous process comprising cell proliferation, differentiation, and division. Much evidence shows that suppressing cell proliferation often results in cell cycle arrest [26,27]. Previous studies found that 20 μM Cd induces cell cycle arrest in the G2/M phase of rat kidney epithelial cells [28]. In addition, Choi et al. (2011) found that exposure to Cd induced cell cycle arrest in the G1 phase in human lung fibroblasts [29]. However, the Cd toxicity to human skin cells is largely unknown. Currently, studies suggested that heavy metals absorption through human skin induced skin cancer [30,31,32], this indicates that heavy metal-induced skin damage may confer enormous health risks to humans. Therefore, it is important to improve the understanding of the detrimental effects of Cd on the skin, especially the underlying mechanisms.

Many studies have shown that Cd can induce apoptosis through various molecular mechanisms [22,23,24]. Pfeffer et al. (2018) found that the apoptosis pathway is activated by intracellular and extracellular signals [33]. From the different initiation stages, apoptosis is often divided into three pathways, namely the mitochondrial pathway, the endoplasmic reticulum (ER) stress pathway, and the death receptor pathway. ER stress may serve as a key mechanism for Cd-induced cytotoxicity [34,35]. Exogenous inducers trigger ER stress to induce apoptosis. However, most studies on Cd-induced apoptosis currently focus on the mitochondria-induced pathway and death receptor pathway, while the involvement of the ER-stress pathway in Cd-induced toxicity is rarely studies.

In this study, to better understand Cd-induced toxicity to human skin, the cell viability, DNA damage, cell cycle arrest, and apoptosis were evaluated in normal human skin keratinocytes (HaCaT) exposed to Cd for 24 h in. In addition, the expression of ER stress genes (*XBP-1*, *BiP*, *ATF-4*, and *CHOP*) was determined to explore the underlying molecular mechanisms associated with Cd-induced adverse effects.

## 2. Results and Discussion

### 2.1. Cd Decreased Cell Viability and Changed Cell Morphology

**Figure 1:**
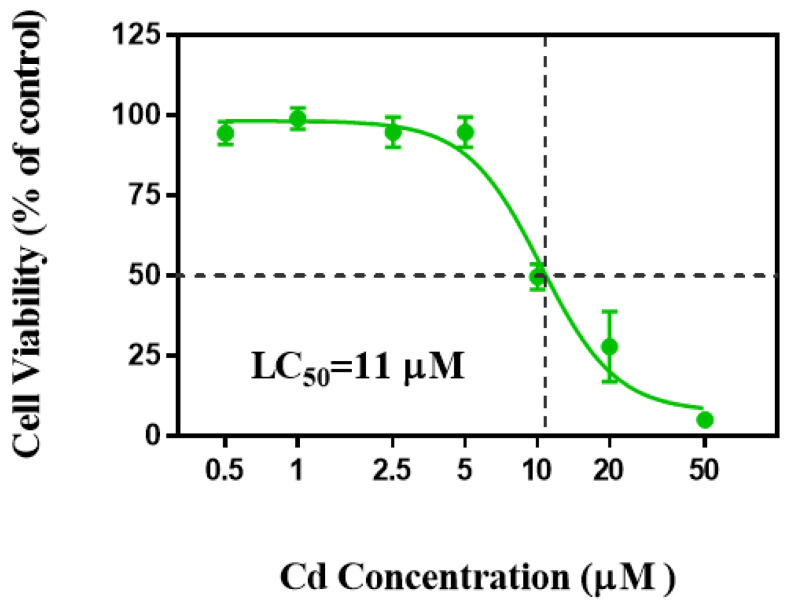
Cell viability of HaCaTs after exposure to Cd for 24 h. The 50% lethal concentration (LC_50_) of Cd was calculated through a nonlinear regression curve (log (inhibitor) vs. normalized response−variable slope).

Cell viability is an important indicator of cytotoxicity and can reflect cellular metabolism and cell death [36]. In this study, we found that the viability of HaCaT cells was not changed until exposure to >5 µM Cd (Figure 1). HaCaT cell viability was inhibited by 47% after exposure to 10 µM Cd and by 72% for 20 µM Cd (Figure 1). Chen et al. (2016) demonstrated the cell viability of vascular endothelial cells (HUVECs) was reduced to 65% after exposure to 40 µM Cd for 24 h [37]. Conversely, Liang et al. (2021) found that exposure to 10 µM Cd promoted the proliferation of breast cancer cells [9]. The results suggest that human skin epidermal cells were more sensitive to Cd than human vascular endothelial cells. Consistent with our results, the viability of A549 cells was significantly decreased by 10 µM Cd compared with the control [38]. Based on cell viability, the LC_50_ of Cd for HaCaT cells was 11 µM, as determined by linear regression analysis (Figure 1), which was lower than that for human gastric epithelial cells (17 µM) [39]. These differences could be attributed to different cells having different tolerance to Cd stress.

**Figure 2:**
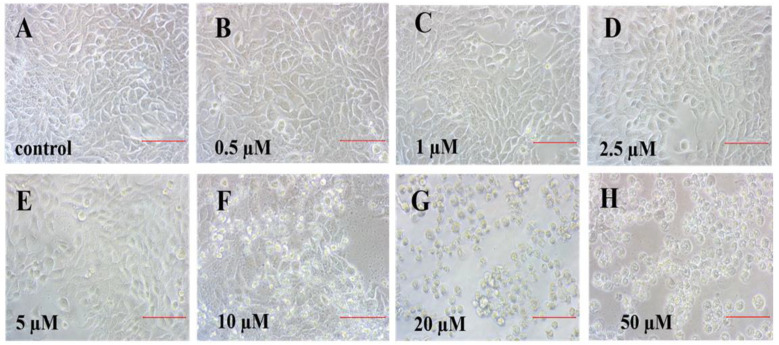
Morphology of HaCaT cells after exposure to Cd at 0 (**A**), 0.5 (**B**), 1 (**C**), 2.5 (**D**), 5 (**E**), 10 (**F**), 20 (**G**), and 50 (**H**) μM for 24 h. Images were recorded using inverted phase-contrast microscopy at 200× magnification (bar = 100 μm).

Cell morphology is also an important indicator of cytotoxicity. In this study, the changes in cell morphology were consistent with cell viability changes (Figure 2A–H). The typical cobblestone and polygonal appearance of a confluent monolayer of HaCaT cells were clear in the control (Figure 2A) and cells exposed to <2.5 µM Cd (Figure 2B–D), whereas HaCaT cells exposed to 5 µM Cd changed from polygonal to oval, with a loose and irregular morphology, and the appearance of floating cells (Figure 2E). As Cd concentration increased, the number of floating cells increased dramatically, and the number of viable cells declined (Figure 2F–H). These data are similar to our previous study, in which the typical cobblestone and polygonal appearance of human gastric epithelial cells was reversed and floating cells in the medium were found after exposure to >20 µM Cd [39]. Thus, to elucidate the underlying mechanisms, we selected 2.5, 5, and 10 µM Cd (lower than the LC_50_) for the subsequent experiments.

### 2.2. Cd Exposure Caused DNA Damage

**Figure 3:**
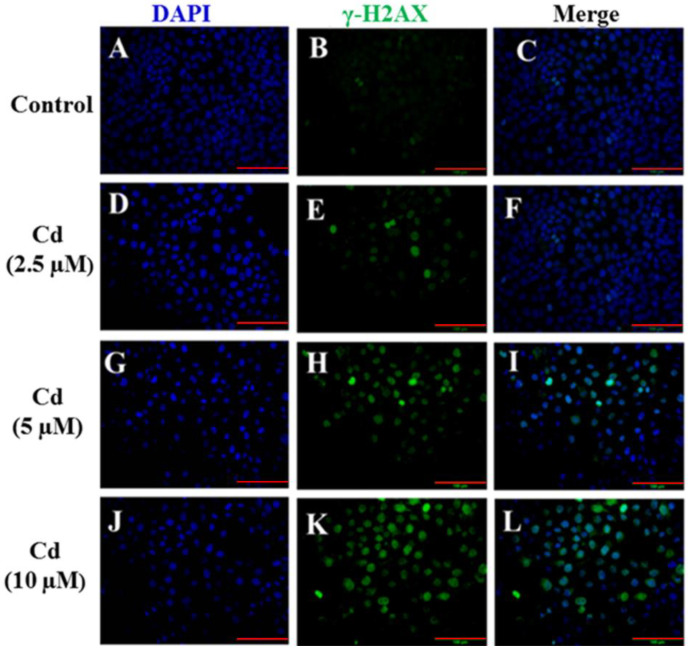
Cd induced DNA damage in HaCaT cells. Cells were exposed to 2.5–10 µM Cd for 24 h. The immunofluorescent staining of γ-H2AX (green: **B,E,H,K**) was used to detect DNA damage in HaCaT cells, and the cell nuclei were labeled with DAPI (blue: **A,D,G,J**). The images were merged using Image-Pro Plus 6.0 software (**C,F,I,L**). (scale bars: 100 μm).

To further substantiate our hypothesis that Cd-induced DNA damage in HaCaT cells, immunohistochemical staining for γ-H2AX, a marker of DNA damage [40,41,42], was evaluated in HaCaT cells after exposure to Cd for 24 h (Figure 3). Phosphorylated histone H2AX (γ-H2AX) is a core player in the DNA damage response (DDR) and serves as a biomarker for DNA double-strand break repair [43,44,45]. DNA damage is often visualized by the formation of γ-H2AX foci in the damaged nucleus [46,47]. The results indicated a significant increase in H2AX phosphorylation focal points after exposure to Cd compared with the control group (Figure 3B,E,H,K), which was consistent with Ou et al. (2021) who showed that Cd induced H2A phosphorylation in a dose-dependent manner in skeletal cells [48]. Similarly, Cd at 1–5 µg/mL caused DNA single-strand breaks in HepG2 cells and there is a gradual concentration–response relationship [49]. In addition, other studies have shown that Cd can cause oxidative DNA damage to sperm in human seminal plasma and lead to decreased sperm quality [50]. The data show that DNA damage was consistent with cell viability (Figure 1) and cell morphology (Figure 2), suggesting that Cd induced cytotoxicity in a dose-dependent manner in HaCaT cells.

### 2.3. Cd Exposure Induced Cell Cycle Arrest and Apoptosis

**Figure 4:**
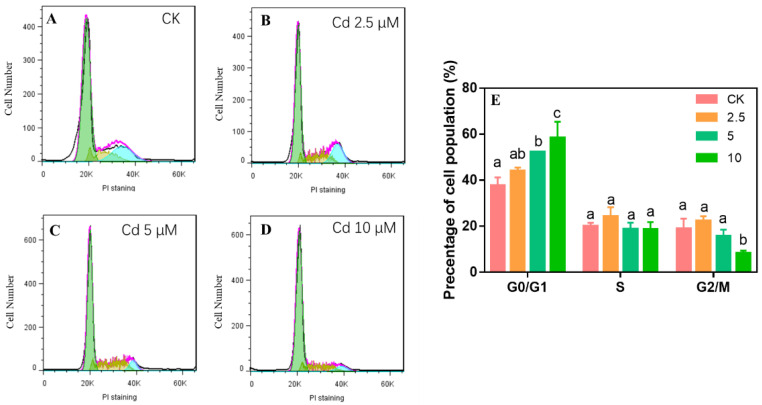
HaCaT cell cycle arrest was induced by exposure to 2.5–10 µM Cd for 24 h (**A**–**E**). Flow cytometry results showing that cells were arrested in G0/G1 phase (**A**–**D**) and the histogram of cell cycle distribution (**E**). Different letters in the same phase indicate significant differences at *p* < 0.05.

To investigate whether Cd caused cell cycle arrest in HaCaT cells, we analyzed the cell cycle distribution after exposure to 2.5–10 µM Cd. The data show that Cd exposure caused cell cycle arrest at the G1 phase in HaCaT cells (Figure 4), which is consistent with Choi et al.’s results that Cd induced cell cycle arrest at the G1 phase in human lung fibroblasts [29]. HaCaT cells (52–58%) were arrested in the G0/G1 phase after exposure to 5–10 µM Cd; this was a higher percentage than that of the control group (15–21%, Figure 4). In addition, after exposure to 10 µM Cd, 8 % of HaCaT cells were in the G2/M phase compared to 19% in the control group (Figure 4). The results showed that Cd triggered cell cycle arrest in the G0/G1 phase in HaCaT cells, which was similar to a previous study in which the cell cycle was arrested at the G0/G1 phase in rat renal tubular epithelial cells exposed to 5 µM Cd [51].

**Figure 5:**
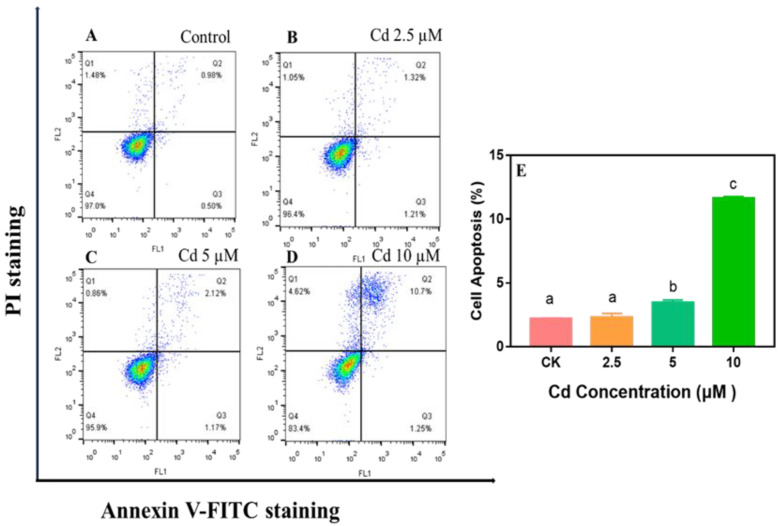
Apoptosis of HaCaT cells was measured using Annexin V-FITC/PI staining by flow cytometry after exposure to 2.5–10 µM Cd for 24 h (**A**–**D**). The statistical analysis graph presents cell apoptosis rate (Q2 + Q3 quadrant) (**E**). Different letters above the columns indicate a significant difference (*p* < 0.05).

Apoptosis is an essential form of cell death [52]. In addition to changes in cell viability, DNA damage, and cell cycle arrest, apoptosis also occurred in HaCaT cells after Cd exposure (Figure 5); this is similar to Wang et al.’s report that Cd induced mouse skin fibroblast apoptosis [27]. Compared with the control group, the proportion of apoptotic cells after Cd exposure increased in a dose-dependent manner. In Figure 5A–D, as the Cd concentration increased, the proportion of cells in the Q2/Q3 region increased, especially at Cd > 2.5 µM (Figure 5C,D). The proportion of HaCaT cells in the Q2/Q3 region was 3.5–12% when Cd was increased to 5–10 µM, which was 1.4–4.8-fold higher than the control group, suggesting that Cd might cause early apoptosis. Compared to controls, HaCaT cell apoptosis increased slightly at 5 µM and dramatically at 10 µM (*p* < 0.05) (Figure 5E), indicating that high concentrations of Cd caused more severe damage in HaCaT cells.

Besides, Xu et al. (2021) pointed out that Cd significantly promoted the apoptosis of KGN cells in a dose- and time-dependent manner [53] However, unlike our study, Cd induced apoptosis at 2.5 μM in KGN cells, which may be due to the different tolerance to Cd in different cells. Taken together, the results showed that apoptosis was associated with Cd-induced cytotoxicity in HaCaT cells.

### 2.4. Cd Exposure Altered ER Stress Gene Expression

**Figure 6:**
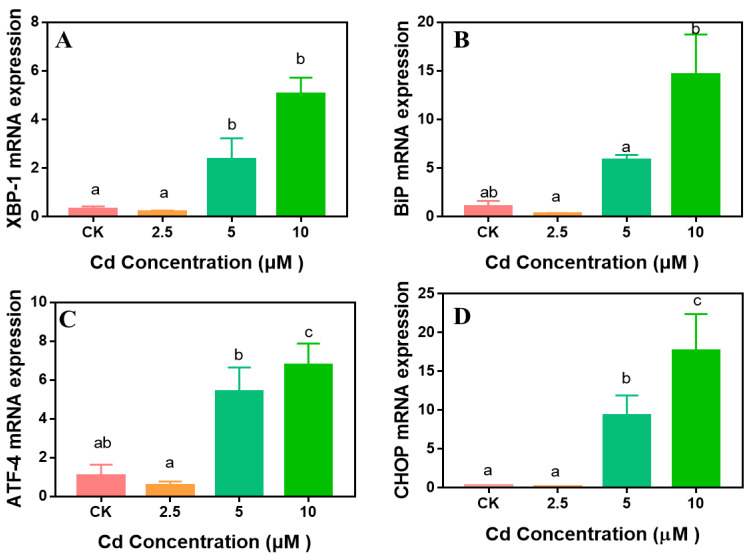
Expression of endoplasmic reticulum (ER) stress-related genes (**A**–**D**) in HaCaT cells after exposure to 2.5–10 µM Cd for 24 h. Results are presented as mean ± SE of three independent experiments. The different letters above the columns indicate a significant difference at *p* < 0.05.

ER stress is an important pathway for Cd-induced cytotoxicity [54]. To better understand the associated molecular mechanism of HaCaT apoptosis, we hypothesized that ER stress may be involved in Cd-induced HaCaT apoptosis. This was based on many previous studies showing that Cd induced the upregulation of ER stress-related genes in HK-2 human renal proximal tubular cells and human bronchial epithelial cells [55,56]. Disruption of the ER protein system by toxicants leads to the accumulation of unfolded proteins in the ER lumen, leading to ER stress. In the ER stress-related signal pathway, *XBP-1* plays an important role; it binds to the unfolded protein response (UPR) promoter elements to initiate the expression of genes that enhance the ER’s ability to process the UPR, including molecular chaperones (*BiP*) and C/EBP homologous protein (*CHOP*) [57]. In this study, we found that the mRNA levels of *XBP-1*, *BiP*, *ATF4,* and *CHOP* were increased by 4–5-, 5–13-, 4–6-, and 9–17-fold after exposure to 5–10 μM Cd for 24 h (Figure 6), respectively, which is consistent with the results that Cd can promote the expression of the ER stress marker *BiP* and the ER stress-associated pro-apoptotic transcription factor *CHOP* in cardiomyocytes [58]. These studies support our hypothesis that ER stress was involved in Cd-induced HaCaT apoptosis.

## 3. Materials and Methods

### 3.1. Chemicals and Reagents

Cd chloride (CdCl_2_, purity 98%) was from Sinopharm Chemical Reagent Co., Ltd. (Shanghai, China). Minimum essential medium (MEM), fetal bovine serum (FBS), penicillin/streptomycin (PS), and 0.25% trypsin-EDTA solution were from Procell Life Science & Technology Co., Ltd. (Wuhan, China). Cell counting kit-8 (CCK-8) cell viability assay kit, Annexin V-FITC/PI apoptosis detection kit, cell cycle analysis kit, and total RNA isolation kit were purchased from Yi Fei Xue Biotech. Co., Ltd. (Nanjing, China). Rabbit monoclonal antibody anti-gamma H2A.X (γH2A.X) was from Abcam (Cambridge, UK).

### 3.2. Cell Culture and Cd Treatment

Human skin keratinocytes (HaCaT) were from American Type Culture Collection. The HaCaT cells were cultured in MEM supplemented with 10% FBS and 1% penicillin–streptomycin solution in an incubator with 5% CO_2_ at 37°C. Before cell treatment with Cd, HaCaT cells were seeded in 6/96-well plates overnight.

### 3.3. Cell Viability Assay

To determine the cytotoxicity of Cd, HaCaT cells were seeded in 96-well plates at a density of 1 × 10^4^ cells/100 μL/well and incubated overnight. The cells were then treated with different concentrations of Cd (0–50 µM) for 24 h. After exposure, 10 μL CCK-8 solution was added to each well, and the cells were incubated at 37 °C for 2 h. The absorbance was measured at 450 nm via a microplate reader (Molecular Devices LLC, San Jose, CA, USA). Then, the cell morphology was observed and recorded by an inverted microscope (TS-100, Nikon, Japan).

### 3.4. Immunofluorescence Staining

Direct immunofluorescence detection was employed for the determination of Cd-induced DNA damage in HaCaT. Briefly, HaCaT cells were seeded into 24-well plates at 5 × 10^4^ cells/well for 24 h. Based on a preliminary experiment, the median lethal concentration (LC_50_) was 11 µM; given that, in this study, the cells were exposed to 2.5–10 µM Cd for 24 h. Then, the cells were scrubbed three times with PBS, fixed with 4% paraformaldehyde for 30 min, and then permeated with 10% Triton X-100 solution at room temperature for 15 min. After being rinsed with PBS three times, the cells were sealed with 1% bovine serum albumin (BSA) for 60 min and then incubated with rabbit anti-γ-H2AX monoclonal antibody (ab81299, Abcam, Cambridge, MA, USA) at 4°C overnight. Subsequently, the cells were rewarmed at room temperature for 1 h, then Goat Anti-Rabbit IgG (H + L) Fluor 488-conjugated antibody was added (Affinity Biosciences, Jiangsu, China), and the cells were incubated at room temperature for 1 h in the dark. Nuclei were counterstained with 4′,6-diamidino-2-phenylindole (DAPI, Yi Fei Xue Biotech. Co., Ltd., Nanjing, China). in the dark for 10 min. Immunofluorescent images were photographed using an inverted microscope system (IX73, Olympus, Tokyo, Japan).

### 3.5. Cell Cycle and Apoptosis Assays

Cell cycle analysis kits were employed to detect the cell cycle distribution. The cells were cultured in a 6-well plate and exposed to 2.5–10 µM Cd for 24 h. Subsequently, HaCaT cells were harvested with 0.25% trypsin-EDTA solution digestion, washed twice with ice-cold PBS, and fixed in ice-cold 70% ethanol overnight at 4 °C. The fixed cells were centrifuged and washed twice with cold PBS. Then, 10 µL RNaseA solution was added and stained with 5 µL propidium iodide (PI). Approximately 1 × 10^4^ cells were loaded onto a CyFlow® Cube16 Flow cytometer (Sysmex Partec, Nuremberg, Germany) to evaluate the distribution of the cell cycle. The cells in different cell cycle phases were analyzed by FlowJo Version 7.6. software (BD Biosciences, Franklin Lakes, NJ, USA).

In addition, for cell apoptosis analysis, HaCaT cells were seeded into a 6-well plate at a density of 1 × 10^6^ cells/mL and were exposed to 2.5–10 µM Cd for 24 h. The cell apoptosis was detected using Annexin V-FITC/PI Apoptosis Detection Kit. Briefly, the cells were harvested by 0.25% trypsin-EDTA solution digestion, washed twice with ice-cold PBS, and re-suspended in 500 µL 1× binding buffer. Then, cell suspensions were mixed with 5 µL Annexin V-FITC and 2 µL PI for 15 min at room temperature in the dark. Finally, PBS was added to the total volume of 1,500 µL. The stained cells were detected using CyFlow® Cube16 Flow cytometer to assess cell apoptosis, and 10,000 events were acquired for each sample. Cells in early apoptosis are only stained by Annexin V-FITC. However, necrotic or late apoptotic cells are positive for both Annexin V-FITC and PI staining. The cell apoptosis ratio was analyzed using FlowJo Version v10. Software (BD Biosciences, Franklin Lakes, NJ, USA).

### 3.6. RNA Extraction, cDNA Synthesis, and Quantitative RT-PCR

We further study the related mechanism of Cd-induced cytotoxicity by detecting the ER-related regulatory genes. In brief, HaCaT cells were treated with 2.5–10 µM of Cd at 37 °C for 24 h, and the total RNA from the cells was extracted using the total RNA isolation kit according to the manufacturer’s protocol. The concentration and quality of RNA were determined using a NanoPhotometer^®^ N60 (IMPLEN GmbH, Munich, Germany). Quantitative real-time PCR (qRT-PCR) was performed to detect ER stress-related gene expression levels (*XBP-1, BiP, ATF-4, CHOP*) by applying SYBR Green qPCR Master Mix and Roche Light Cycler. The reaction cycler conditions were 95 °C for 10 min at first, and then 40 cycles of 95 °C for 15 s and 60 °C for 1 min. After the last cycle, melting curves were obtained from 65 to 97 °C with an increment of 0.5°C every second to ensure reaction specificity. The data were calculated by the 2^−ΔΔCT^ method, with the relative expression level of targeted genes being normalized to the reference gene, β-actin. The primer sequences are listed in Table 1.

### 3.7. Statistical Analysis

All experiments were repeated three times. The data are expressed as means ± SE. Statistical analysis was conducted using one-way ANOVA with Tukey multiple comparisons (Tukey–Kramer) test by GraphPad Prism Version 7.0 software (GraphPad Software LLC, CA, USA). A *p* value of <0.05 was regarded as statistically significant.

## 4. Conclusions

In this study, we confirmed a concentration-dependent decrease in the cell viability was induced by exposure to 0–50 μM Cd for 24 h, with an LC_50_ of 11 μM. Moreover, Cd-induced DNA damage was evidenced by upregulation of the level of γ-H2AX. In addition, Cd induced G0/G1 phase cell cycle arrest and apoptosis in a dose-dependent manner, and upregulated the mRNA levels of ER stress-related genes *(XBP-1*, *BiP*, *ATF4*, and *CHOP*). In summary, our results demonstrated that Cd exposure induced cytotoxicity and DNA damage, which resulted in cell cycle arrest and apoptosis in HaCaT cells. The ER stress signal pathway is involved in Cd-induced apoptosis of HaCaT cells.

## Figures and Tables

**Table 1:** Primers for RT-qPCR of ER stress regulatory genes.

Gene	Forward Primer (5′-3′)	Reserve Primer (5′-3′)	Accession No.	Production Size (bp)
*XBP-1*	TTACGAGAGAAAACTCATGGCC	GGGTCCAAGTTGTCCAGAATGC	NM_005080.3	283
*BiP*	CACGGTCTTTGACGCCAAG	CCAAATAAGCCTCAGCGGTTT	NM_005347.4	215
*ATF4*	ATGACCGAAATGAGCTTCCTG	GCTGGAGAACCCATGAGGT	NM_182810	153
*CHOP*	GGAAACAGAGTGGTCATTCCC	CTGCTTGAGCCGTTCATTCTC	NM_001195055	116
β-actin	GTACCACTGGCATCGTGATGGACT	CCGCTCATTGCCAATGGTGAT	NM_001101.3	323

## Data Availability

The data are all presented in this study.
